# Emerging Infectious Diseases Laboratory Fellowship Program.

**DOI:** 10.3201/eid0103.950313

**Published:** 1995


- Solicit and allocate specific resources to deal with the emerging diseases initiative, both at the regional and country levels. A portion of these funds should be immediately available when outbreaks are recognized.

For more information on these recommendations, the conference, or its plan of action, contact PAHO.

**Daniel B. Epstein**
Office of Information & Public Affairs
Pan American Health Organization
Washington, D.C., USA

### Emerging Infectious Diseases Laboratory Fellowship Program

A partnership has been established between the Association of State and Territorial Public Health Laboratory Directors and the Centers for Disease Control and Prevention (CDC) to develop and initiate an emerging infectious diseases laboratory fellowship program in January 1996. A goal of this fellowship program is to strengthen local, state, and federal public health infrastructures to support surveillance and implement prevention and control programs. The fellowship program will help recruit and train microbiologists for laboratories nationwide and provide opportunities for doctoral level scientists to conduct high-priority infectious disease research.

The emerging infectious diseases fellowship program will offer a 2-year laboratory research track for doctoral level scientists, with emphasis on applied research or development in infectious diseases and a 1-year advanced laboratory training track for bachelor's and master's level scientists, with emphasis on the practical application of emerging infectious diseases technologies, methods, and practices. Fellow training and research will take place at CDC and state and local public health laboratories.

For applications or additional information, contact

**Emerging Infectious Diseases Fellowship Program**
**Association of State and Territorial Public Health Laboratory Directors**
**1211 Connecticut Avenue, Suite 608**
**Washington, D.C. 20036**
**Phone: 202-822-5227, Fax: 202-887-5098**

### Tenth Annual ASTPHLD Conference on Human Retrovirus Testing

The Tenth Annual Conference on Human Retrovirus Testing, sponsored by the Association of State and Territorial Public Health Laboratory Directors (ASTPHLD), was held March 6 to 9, 1995, in Reno, Nevada. The conference, which was attended by more than 300 representatives of public and private sector laboratories as well as test kit manufacturers, emphasized three themes: new human immunodeficiency virus (HIV) variants, international issues, and HIV testing of newborns. The topics discussed included sequence data for type O isolates, the search for new HIV variants, zidovudine (AZT) resistance, decreased maternal-neonatal transmission due to AZT prophylaxis, results of the national anonymous survey of HIV prevalence in the United States, and the ethical concerns of perinatal screening.

An international perspective on HIV testing was brought to the conference by presentations that focused on India and Latin America. Results were given of a project, funded by a 12-month study grant from the World AIDS Foundation, to provide training on HIV testing to laboratories in India. Four Indian facilitators were trained in the United States; they provided translation and other assistance to eight ASTPHLD faculty, who gave workshops in four training centers in India. This training, which focused on enzyme immunoassay, linked trainees with staff from Indian reference centers and established training materials and trainers for future workshops to be conducted by Indian staff.

Laboratory aspects of HIV testing in Latin American and the Caribbean were also discussed by a member of the Pan American Health Organization (PAHO), who described the spectrum of HIV incidence rates and testing algorithms. PAHO is asking countries of the region to assess their algorithms in terms of sensitivity, specificity, and cost. PAHO aims to support national laboratories by providing guidelines and quality assurance. Proficiency testing, which is encouraged, will be provided by the Centers for Disease Control and Prevention.

ASTPHLD's 11th Annual Human Retrovirus Conference is set for March 6-8, 1996, in Orlando, Florida. Requests for additional information are available; FAX request to 202-887-5098.

**James L. Pearson**
Division of Consolidated Laboratory Services,
Commonwealth of Virginia, Richmond, Virginia, USA

